# Usefulness of Direct Auricular Artery Injection as Refinement of the Well-Established Rabbit Blood Shunt Subarachnoid Hemorrhage Model

**DOI:** 10.3390/brainsci15080826

**Published:** 2025-07-31

**Authors:** Stefan Wanderer, Michael von Gunten, Daniela Casoni, Stefano Di Santo, Jürgen Konczalla, Ali-Reza Fathi

**Affiliations:** 1 Department of Neurosurgery, Kantonsspital Aarau, 5001 Aarau, Switzerland; 2 Program for Regenerative Neuroscience, Department for BioMedical Research, University of Bern, 3008 Bern, Switzerland; stefano.disanto@insel.ch; 3 Neurochirurgie Fathi AG, Schachen 22, 5001 Aarau, Switzerland; fathi@hin.ch; 4 Institut für Pathologie, Länggasse, 3063 Ittigen, Switzerland; vongunten@patholaenggasse.ch; 5 Experimental Surgery Facility, Formerly Under the Department for Biomedical Research, Faculty of Medicine, University of Bern, 3008 Bern, Switzerland; daniela.casoni@dbmr.unibe.ch; 6 Department of Neurosurgery, Johann Wolfgang Goethe-University, 60528 Frankfurt am Main, Germany; j.konczalla@med.uni-frankfurt.de

**Keywords:** animal models, auricular artery, digital subtraction angiography, subarachnoid hemorrhage, refinement

## Abstract

Introduction: Given the impact of aneurysmal subarachnoid hemorrhage (aSAH) on patients’ health, preclinical research is substantial to understand its pathophysiology and improve treatment strategies, which necessitates reliable and comprehensive animal models. Traditionally, aSAH models utilize iliac or subclavian access for angiography, requiring invasive procedures that are associated with significant risks and animal burden. This pilot study explores a less invasive method of digital subtraction angiography (DSA) by using the auricular artery (AA) as an alternative access point. Our aim was to demonstrate the feasibility of this refined technique, with the intention of reducing procedural risks, providing shorter operation times with enhanced neurological recovery, and simplifying the process for both researchers and animals. Materials and Methods: In this study, six female New Zealand white rabbits (3.2–4.1 kg body weight) underwent experimental induction of aSAH via a subclavian-cisternal shunt. The initial steps of this procedure followed traditional techniques, consisting of subclavian access through microsurgical preparation, followed by DSA to analyze retrograde filling of the basilar artery (BA). To evaluate the alternative method, on day 3 after induction of aSAH, DSA was performed via the AA instead of the traditional subclavian or femoral access. A catheter was placed in the AA to allow retrograde filling of the BA. This approach aimed to simplify the procedure while maintaining comparable imaging quality. Results: All rabbits survived until the study endpoint. Postoperatively, two rabbits showed signs of hemisyndrome, which significantly improved by the time of follow-up. No additional morbidities were observed. Upon euthanasia and necropsy, all animals showed clear subarachnoid bleeding patterns. DSA via the AA produced strong contrasting of the BA comparable to the traditional method. Conclusions: This technical note presents an initial evaluation of AA access as a feasible and potentially advantageous method for DSA in a rabbit model of blood shunt subarachnoid hemorrhage. The method shows promise in reducing invasiveness and procedural complexity, but further studies are required to fully establish its efficacy and safety. Future research should focus on expanding the sample size, refining the anatomical understanding of the AA, and continuing to align with ethical considerations regarding animal welfare.

## 1. Introduction

Aneurysmal subarachnoid hemorrhage (aSAH) represents a hemorrhagic type of stroke caused by rupture of an intracranial aneurysm, with blood distribution into the subarachnoid space. Risk factors for aneurysm bleeding include hypertension, smoking, excessive alcohol consumption, and genetic predispositions such as polycystic kidney disease and connective tissue disorders [1]. Symptoms, often described as sudden and severe headaches, can occur abruptly, and are often associated with prior warning leakages. This sudden bleeding onset usually leads to rapid neurological deterioration. ASAH itself is associated with various complications, such as potential rebleeding, delayed cerebral vasospasm (DCVS), hydrocephalus, cortical spreading depressions, and delayed cerebral ischemia (DCI), a multifactorial process responsible for poor overall patient outcomes [2].

Approximately 25% of patients with aSAH die within 24 h, and about 40–50% die within a month despite medical intervention. Many survivors are left with significant neurological deficits [3].

aSAH research has made significant progress over time, focusing on early diagnosis, improved treatment options, and better understanding of the underlying pathomechanisms [4,5]. Endovascular coiling and microsurgical clipping techniques are both valid treatment options [6,7,8]. Studies on genetic factors contributing to aneurysm formation and rupture have opened new ways of identifying individuals at risk and developing targeted preventive strategies [9,10,11,12,13,14].

However, robust animal models with a multimodal analytical approach remain crucial to gaining better insights into treating aSAH-associated sequelae and ameliorating patient outcomes. In this context, the closed-cranium rabbit blood shunt subarachnoid hemorrhage model has been developed to address the limitation of only focusing on DCVS rather than accurately mimicking the acute events of aneurysm rupture. This model allows researchers to simulate both early brain injury and DCVS, providing a more comprehensive understanding of aSAH’s pathophysiology [13,14,15,16,17,18]. So far, traditional aSAH models imply gaining iliac or subclavian access for bleeding induction. Therefore, the aim of our study was to explore a comparable and less invasive injection technique via auricular artery (AA) access using the well-established closed-cranium rabbit shunt model.

## 2. Methods

### 2.1. Animals and Brief Study Design

Six female New Zealand white rabbits, aged 12 weeks and with a body weight of 3.2–4.1 kg, were allocated to the aSAH group. In prior experiments, animals were kept socially in rooms with controlled temperature (20 ± 2 °C) and 50% humidity), under a 12 h day–night cycle. Food pellets and tap water were provided without restriction.

All surgical procedures were performed in the Experimental Surgery Facility (ESF), University of Bern, Switzerland. In this refined closed-cranium rabbit shunt model, digital subtraction angiography (DSA) and bleeding induction were performed via extracorporeal shunting by connecting the arterial catheter, placed in the subclavian artery (SA), to a spinal needle placed into the cisterna magna.

### 2.2. Experimental Protocol

#### 2.2.1. Animal Preparation: Positioning and SA Cannulation on Day 1

**Figure 1:**
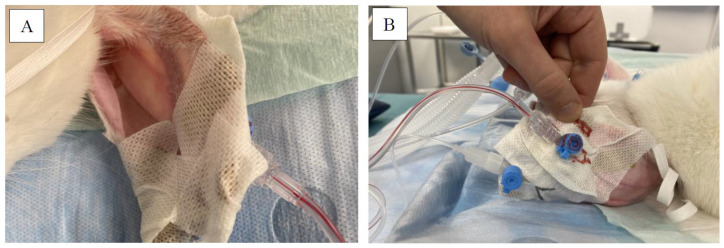
Showing the rabbit in (**A**) supine and (**B**) prone position on the operation table, already intubated with an arterial (**A**) line placed medially in the left AA and venous line (**B**) placed laterally in the left auricular vein.

After clinical examination, as well as weighing and rubbing EMLA cream on the outer auricular surface, sedation was achieved with subcutaneous (SC) injection of ketamine HCl (20 mg/kg) (Narketan 100 mg/mL) and xylazine (6 mg/kg) (Xylapan 20 mg/mL). The animals were allowed to be undisturbed for at least 10 min in an on-purpose box. Thereafter, oxygen was supplied via a mask at a rate of 2–4 L/min and sedation was evaluated after further 5 min. Then, all rabbits were lifted onto a table under oxygen supplementation, followed by clinical and instrumental monitoring (pulse oximeter with plethysmography). A 22–24 G catheter was introduced into the AA, and a 22–24 G cannula was inserted into the marginal auricular vein (see Figure 1).

Both eyes were lubricated with Vitamin A ointment to prevent dryness and irritation. The surgical area was clipped, shared, and disinfected.

An intravenous (IV) bolus of midazolam (0.2–1 mg/kg) and, if necessary, propofol (1–6 mg/kg) was administered to achieve an adequate depth of anesthesia to perform endotracheal intubation. Fluid therapy consisted of Ringer’s lactate (5 mL/kg/h). During the procedure, clinical and instrumental monitoring was assured and consisted of 3 peripheral ECG leads, pulse rate and oxygen saturation, blood pressure (both invasive and non-invasive with Doppler technique), rectal temperature and gas fractions (O_2_, CO_2_, isoflurane), along with processed values of depth of anesthesia via BIS monitoring. All data have been recorded electronically for further assessment.

General anesthesia was maintained with isoflurane in O_2_ (targeting EtIso 1.3%), and analgesia was provided through continuous infusion rate (CRI) of fentanyl (5–20 mcg/kg/h). Nociception was continuously monitored through autonomic responses (blood pressure, respiratory rate, heart rate). If antinociception was deemed inadequate (one of the autonomic variables increased by at least 20% with respect to the baseline), additional boluses of fentanyl or ketamine (1 mg/kg) were provided and the CRI of fentanyl increased in steps of 5 mcg/kg up to a maximum dose of 20 mcg/kg/h. In particular, ketamine was given if the depth of anesthesia was deemed insufficient, based on monitoring of EEG and jaw tone. Normothermia was ensured through an air-forced warming device. Arterial blood gas analysis was conducted at least once during the surgical procedure. Spontaneous ventilation was supported to achieve normocapnia (p_a_CO_2_ 35–45 mmHg) or substituted by pressure-limited, volume-guaranteed mechanical ventilation if necessary. Rabbits were positioned in dorsal recumbency slightly laterally towards the side where SA would be prepared. After sterile preparation of the skin area above the right pectoral muscle, local anesthetic (Ropivacaine 0.5%, maximum 3 mg/kg) was infiltrated peri-incisionally. A parasternal skin incision was carried out, followed by dissection of the SA under microscope guidance. Proximal and distal ligatures were applied around the exposed end, and one ligature was kept close to the proximal control to secure the catheter. The vessel was ligated distally. An arteriotomy was conducted, and a small intravascular 3-french catheter was placed retrogradely into the SA. The catheter was secured with a double-knot ligature towards the distal ligature to prevent arterial twisting and to avoid slippage and/or massive bleeding.

Hypotension, defined as a mean arterial pressure (MAP) lower than 60 mmHg, was prevented with the use of noradrenaline (0.1–0.4 mcg/kg/min).

#### 2.2.2. Hemodynamics Monitoring: Blood Pressure and Arterial Blood Gas Analysis

A 3-way stopcock was connected between the arterial cannula and the transducer to facilitate arterial blood gas sampling. The transducer was zeroed at the level of the left atrium and its position readapted when the recumbency of the animal was changed.

#### 2.2.3. Establishing Baseline: DSA for Precise Assessment

To ensure accurate measurements, a spherical external sizing device was placed at both mandible angles before performing DSA. A retrograde intra-arterial bolus of non-ionic contrast medium iopamidol (0.6 mL/kg, infused at 5 mL/s for 2 s) was injected through the cannulated artery, followed by immediate saline flushing to prevent occlusion.

#### 2.2.4. Transitioning to Prone Position: Optimal Repositioning

Following baseline DSA, rabbits were carefully turned in sternal recumbency, ensuring that intra-arterial catheters remained in situ. The head was supported at a 30° angle downwards using a head-holder.

#### 2.2.5. Insertion of Intracranial Monitors: Monitoring Pressure and Blood Flow

A midline incision through the skin and galea was made to introduce a small surgical retractor. A burr hole, 5–10 mm in diameter, was created with a high-speed microdrill at predetermined coordinates for placing an intracranial pressure (ICP) probe. The dura was visualized, coagulated, and carefully opened with a scalpel. Hemostasis was achieved using bone wax and bipolar coagulation. The ICP monitor tip was inserted into the right olfactory bulb at a depth of 2 mm and then calibrated.

After probe placement, the burr hole was sealed with bone wax. Baseline measurements of MAP, ICP, and cerebral blood flow were obtained using a multiparameter monitor.

#### 2.2.6. Targeting the Cisterna Magna: Needle Placement

**Figure 2:**
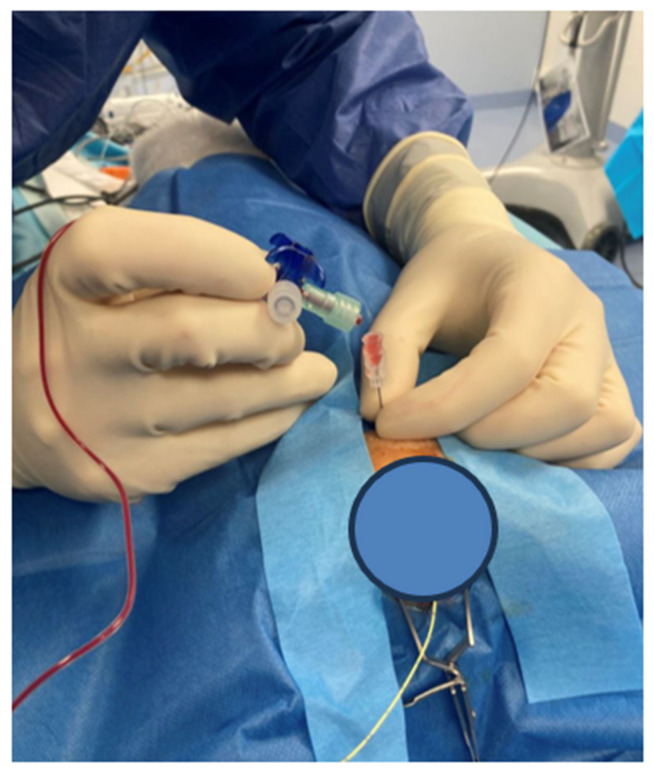
Prior aSAH induction on day 1. The rabbit is positioned in prone position, followed by a sterile draping of the head. An ICP probe (yellow) is placed in the right frontal lobe; a pediatric spine needle inserted into the cisterna magna showing CSF already mixed with a drip of blood from subclavian access.

The surgical field over the head and neck was disinfected twice with povidone–iodine, followed by coverage with sterile sheets. A 22 G × 40 mm pediatric spinal access needle was inserted transcutaneously into the cisterna magna, confirming anesthesia adequacy via lack of response to stimuli. Proper needle placement was confirmed by the presence of cerebrospinal fluid (CSF) in the bevel and was able to drip while the rabbit’s head was positioned 20° down (see Figure 2).

#### 2.2.7. Initiating Shunt Flow: Controlled Pressure Modulation

The spinal access needle was connected to the previously cannulated SA through blood-filled pressure monitoring tubing. The 3-way stopcock served both in pressure measurement and sampling. The severity of aSAH was correlated with the volume of blood, roughly determined by assessing subarachnoid clot extents after brain harvesting. Anesthesia depth and nociception were evaluated before initiating the shunt between the SA and cisterna magna. A controlled aSAH could be achieved by selectively stopping the shunt based on ICP levels. Steady-state values were recorded over approximately 15 min. The spinal needle remained in situ until ICP values stabilized close to baseline. The shunt was closed if the ICP plateau persisted above baseline for longer than 10 s or if a flattening of the EEG occurred spontaneously. The ICP probe was subsequently removed, and the burr hole was sealed with bone wax. All catheters, including the subclavian catheter, were removed due to associated risks with manipulation, which could lead to significant complications.

#### 2.2.8. Angiography via Auricular Access

**Figure 3:**
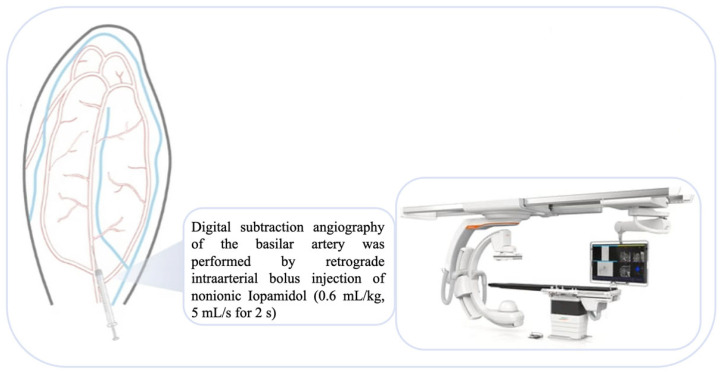
Auricular access was established by catheterization of the AA. DSA was performed by retrograde intra-arterial bolus injection of non-ionic iopamidol (0.6 mL/kg, 5 mL/s for 2 s) through the cannulated artery.

Angiography was performed on day 1 and 3. Instead of using the complex and simultaneously traumatic access variants of subclavian or inguinal access, auricular access (see Figure 3) was chosen.

### 2.3. Postoperative Care Management: Pain Control and Monitoring

The surgical session lasted approximately two hours, followed by the recovery time for rabbits as needed. Tracheal extubation took place when the rabbits were able to swallow and chew spontaneously. Meloxicam 0.5 mg/kg was administered IV to every rabbit. All animals were housed within the ESF for close postoperative observation during the first night, where their neurological status was checked at least every two hours. Supplemental buprenorphine was administered IV (0.02 mg/kg) if the grimace scale was ≥4/10.

To ensure sustained analgesia over the following 72 h, transdermal fentanyl patches (12.5 μg/h) were applied to the outer surface of the ear.

### 2.4. Experimental Day 3: Assessment of Vascular Dynamics

On the third day of the experiment, all steps except insertion of the ICP probe and induction of the extracorporeal shunt were repeated. This repetition ensured the consistency and accuracy of the experimental model. Before euthanasia, a contrast agent was injected via the SA and afterwards via AA. Both administrations aimed to achieve and compare retrograde filling of the BA.

Following the completion of these steps, the animals were euthanized. This process involved administering an IV injection of penthobarbital at a dosage of 1000 mg/kg (Eskonarkon, Streuli Pharma AG, Uznach, Switzerland). When histology and immunohistochemistry were necessary, an intracardiac perfusion-fixation was performed at room temperature at a perfusion pressure of 100 cmH_2_O. This ensured proper preservation of tissue integrity for subsequent analysis and examination.

### 2.5. Ethic Approval and Statistics

The study was performed in accordance with the National Institutes of Health guidelines for the care and use of experimental animals and with the approval of the Animal Care Committee of the Canton of Bern, Switzerland (BE 77/26 October 2021). All surgical procedures were performed under sterile conditions at the ESF (Department for Biomedical Research), University of Bern, Switzerland.

All veterinary care was performed in accordance with the institutional guidelines and conducted under supervision of a board-certified veterinarian anesthesiologist. The ARRIVE guidelines and the 3R principles were strictly followed [10,19].

BA diameters were measured with image-J software (Image-J version 1.52n, U.S. National Institutes of Health, Bethesda, MD, USA, https://imagej.net, accessed on 28 July 2025).

## 3. Results

All of the rabbits included (*n* = 6) in this feasibility study survived until the planned follow-up (FU), resulting in a mortality rate of 0%. Additional data collected included the preoperative weights of the six animals (ranging from 2.5 to 4.2 kg body weight) and their weights before euthanasia (ranging from 2.7 to 3.9 kg body weight).

Records of general morbidity, such as postoperative transient hemisyndrome, and overall mortality rates were also documented. Only two postoperative hemisyndromes have been observed, which significantly improved by the time of the FU. No further relevant morbidities were detected.

**Figure 4:**
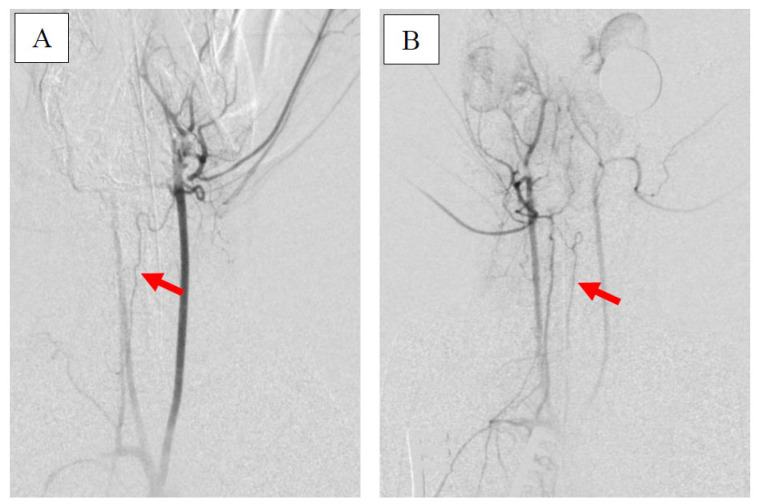
Comparison between left AA (**A**) injection on day 3 and SA (**B**) injection on day 0. The red arrow depicts the course of the BA. Note the spastic cerebrovasculature on day 3.

Furthermore, the diameter of the BA and the presence of DCVS were carefully analyzed on day 3 with a mean diameter of 345 ± 62 µm (Animal 1: 320 µm, animal 2: 330 µm, animal 3: 350 µm, animal 4: 460 µm, animal 5: 270 µm, animal 6: 340 µm). AA injection led to a strong retrograde contrasting of the BA in all animals (see Figure 4A). Imaging quality was comparable for AA and SA injections.

**Figure 5:**
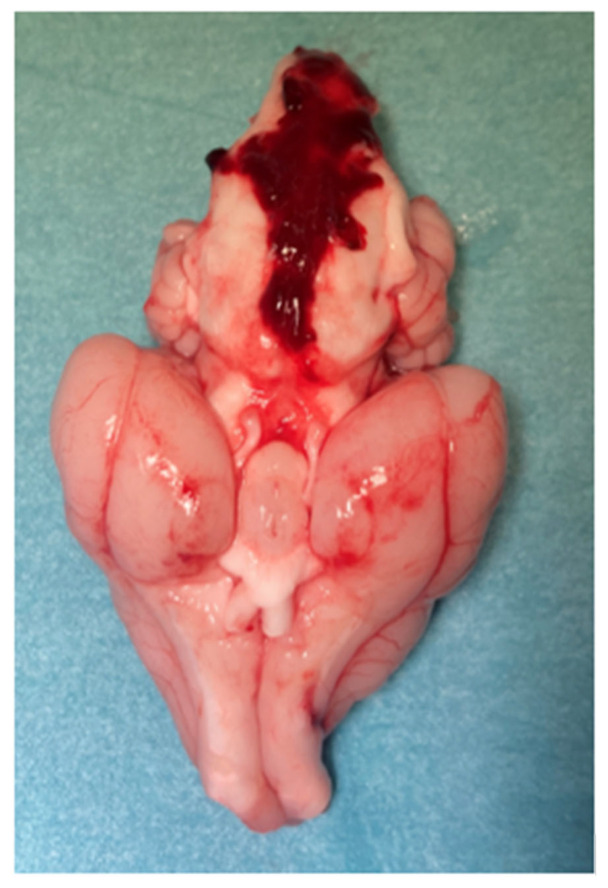
The basal surface of the brain with its vessels is shown depicting strong subarachnoid bleeding pattern with clot formation in the subarachnoid space.

Upon examination, each rabbit displayed a noticeable pattern of bleeding in the subarachnoid space, indicating successful induction of aSAH (see Figure 5).

Additionally, even when not directly measured in this feasibility trial, the sole AA injection would have significantly shortened the overall operation time compared to SA injection. The average preparation time to gain access to the SA by splitting the pectoralis muscle and inserting the tube has been noted as 45 min when performed by an experienced surgeon.

## 4. Discussion

To the best of authors’ knowledge, we demonstrated for the first time that imaging quality assessed in DSA via AA injection is comparable to SA injection. This injection technique depicts an optimization of the DSA technique and, as alternative access via AA, drastically ameliorates conventional invasive methods such as subclavian or iliac access. Furthermore, not only overall operative procedure can significantly be reduced, but even more periprocedural morbidity. Neurological recovery is also facilitated by avoiding splitting of the pectoralis muscle, as potential lesioning of the plexus brachialis is avoided. Auricular access represents therefore an important benefit in terms of animal welfare by adhering to the 3Rs (replacement, refinement, reduction) and therefore helping to minimize periprocedural stress for the animals.

Until recently, intracranial aneurysms present unique challenges in both diagnosis and treatment, due to their location and potential for rupture with life-threatening consequences [20]. While sharing some similarities in pathogenesis and management with aortic aneurysms [21], their anatomical and physiological differences necessitate tailored approaches for accurate assessment and interventions. In research focusing on aneurysm pathobiology using extracranial aneurysm models, intravenous digital subtraction angiography (IVDSA) has been commonly utilized to examine aneurysms originating from the common carotid artery (CCA) in rabbit models. Ding and colleagues noted that IVDSA can effectively substitute intra-arterial DSA for imaging aneurysms created in rabbits [22]. However, Miskolczi et al. highlighted a major drawback in utilizing IVDSA, noting that contrast injected through the ear marginal vein in rabbits becomes heavily diluted, resulting in poor image contrast [23].

Aneurysms in rabbit models are often induced by extracranial aneurysm models via the CCA or SA, making traditional DSA techniques challenging due to the need for surgical exposure of the CCA, SA, or iliacal artery. However, our study shows that DSA via the AA provides clear and precise imaging quality, allowing for accurate measurement of the BA diameter without the need for more invasive surgical procedures. This method offers advantages such as repeatability, as the ear central artery can be cannulated on both sides when needed, can be cannulated more than once, and with minimal post-procedural hematoma, facilitating longitudinal FU studies without the complications associated with surgical access to the CCA, SA, or iliac artery.

Furthermore, the auricular injection technique obviates the need for pectoralis muscle splitting. This elimination of a step not only simplifies and shortens operative procedure, but it also mitigates the risk of postoperative neurological deficits and provides better and faster neurological recovery. Pectoralis muscle splitting can lead to plexus brachialis trauma and subsequent neurological impairment, which is circumvented by the adoption of auricular injection.

The efficacy and advantages of this aSAH model are multifaceted. This study represents the first implementation of an aSAH model utilizing auricular injection in rabbits. Notably, the utilization of this novel technique contributed to the absence of major bleeding and 0% mortality among the rabbits, while also considering other factors like excellent anesthesiological support throughout the whole procedure.

Compared to traditional approaches, such as SA or iliacal injection, the auricular route minimizes the duration of the surgical procedure, thereby decreasing the overall exposure to anesthesia agents. Utilizing DSA via the ear central artery, our results demonstrate the feasibility and efficacy of this minimally invasive technique compared to traditional methods, such as direct measurement with invasive intra-arterial DSA via the CCA or SA. Furthermore, our study demonstrates that the utilization of auricular access for administering contrast agent results in imaging quality comparable to that achieved with carotid or subclavian access, highlighting its potential advantages.

Additionally, the auricular approach eliminates the challenges associated with catheter dislocation when turning the rabbit from dorsal to sternal in the closed-cranium rabbit shunt model. Indeed, traditional approaches may result in catheter displacement during animals’ repositioning, compromising data accuracy and necessitating additional interventions. Contrarily, the use of auricular injection provides stable catheter placement throughout the experiment, thus minimizing the occurrence of complications as mentioned above. Comparing this technique with conventional intra-arterial DSA via CCA or SA injection, these results highlight the benefits of the ear central artery approach, including reduced invasiveness, procedural complexity, operation times, and potential harmful complications. Auricular access itself is, in terms of other already established injection techniques, a less invasive injection technique, directly reducing periprocedural complexity. Microsurgical preparation of the auricular artery is not necessary, in contrast with invasive models such as subclavian and/or femoral access. Therefore, the last two access variants are technically more challenging than simply canulating the auricular artery itself. Furthermore, the surgical risk for auricular injections is low; perioperative morbidity therefore decreased with no risk of deep vessel perforation with relevant blood loss and/or catheter dislocation when turning the rabbit from supine to prone. By using the auricular approach only, operation times, even those not measured in this feasibility study, would be significantly shorter and, therefore, by reducing the anesthesia burden, neurological recovery facilitated (see Table 1).

Furthermore, our study establishes a good correlation between DSA via the ear central artery and conventional intra-arterial DSA, indicating its reliability as an alternative imaging modality for assessing intracranial aneurysms in rabbit models.

In summary, with this refinement via auricular access, we demonstrate a novel, reproducible, and standardized protocol, which may serve as an important tool for further preclinical investigations in terms of aSAH treatment. It offers, as mentioned above, several advantages over traditional approaches. As such, this novel technique represents a significant advancement in the field of experimental aSAH induction, contributing to improved animal welfare and data accuracy in preclinical research. Throughout the study, strict adherence to the principles of replacement, reduction, and refinement (the 3 Rs) in animal research was maintained. This included refining the surgical technique to minimize animal discomfort and distress, reducing the number of animals used through careful experimental design, and continuously seeking improvements to enhance the welfare of the experimental subjects.

By utilizing auricular access only, there is no need for subclavian access, thereby streamlining the procedure and minimizing potential complications associated with catheter dislodgement when changing the rabbit’s position.

As disadvantages and limitations of this study, in terms of a clear and intended feasibility study, the low animal number has to be highlighted. Furthermore, in order to strictly follow the 3R principles regarding animal welfare, this model requires many human as well as other resources (ESF, a veterinarian, a surgical assistant, a nurse, and anesthesia machines).

This experimental feasibility study describes the first application of auricular access in the closed-cranium rabbit subarachnoid hemorrhage model and demonstrates promising results. Nevertheless, this technique has to be analyzed with bigger sample sizes in future investigations to confirm its safety and efficacy.

### Limitations

Besides the lack of quantitative comparison between AA and SA access, this study is strictly a feasibility study, and as such, it has the inherent limitation of a small sample size. With only 6 animals, the statistical power is limited, and therefore, more advanced statistical analyses are not feasible at this stage. Regarding mortality, a bleeding-induced mortality rate of up to 30% would be consistent with previous experimental procedures using this model. Based on prior studies, the expected effect size is approximately 0.75. Using this effect size, along with an alpha level of 0.05 and a power of 80%, a sample size of 18 rabbits would be recommended for more definitive results. Considering the expected mortality rate of around 30% per group, we would need about 3 rabbits per group, which totals approximately 21 rabbits for the study. 

## 5. Conclusions

Our study presents DSA via AA as promising alternative to conventional intra-arterial DSA injection techniques (CCA, SA, iliac) for assessing BA diameters in rabbits and inducing aSAH comparable to the more well-known SA injection model. This minimally invasive technique offers a high success rate and equal imaging quality in visualizing BA diameters via auricular compared to subclavian access. Operative efficiency is improved by providing significant shorter operation times. Furthermore, this less invasive procedure avoids potential complications related to subclavian access (splitting the pectoralis muscle, deep SA perforation) and reduces risk of catheter dislocation when turning the rabbit from supine to prone, providing lowered mortality. Lastly, animal welfare is addressed by strict adherence to the 3R principles.

## Figures and Tables

**Table 1:** Comparison of auricular, subclavian, and femoral access in terms of advantages and disadvantages.

Auricular Access	Subclavian Access	Femoral Access
Less invasive	Invasive	Invasive
Less complex procedure	Complex Procedure	Complex procedure
Decreased perioperative morbidity, no risk of deep vessel perforation with relevant blood loss, catheter dislocation	Increased perioperative morbidity, potential risk of deep SA perforation with relevant blood loss, catheter dislocation when turning the rabbit from supine to prone	Increased perioperative morbidity, potential risk of deep femoral artery perforation with relevant blood loss, catheter dislocation when turning the rabbit from supine to prone
Significant shorter operation times	Prolonged operation time	Prolonged operation time
Facilitated neurological recovery by avoiding splitting muscles	Potential neurological impairment by splitting the pectoralis muscle	Potential neurological impairment by femoral nerve damage

## Data Availability

The authors declare that all supporting data are available within the article.
